# International Medical Annual and Practitioner’s Index for 1890

**Published:** 1890-05

**Authors:** 


THE INTERNATIONAL MEDICAL ANNUAL AND PRACTITIONER'S INDEX FOR 1890. Edited by P. W. Williams, M. D., Secretary of Staff, assisted by a corps of thirty-six collaborators—European and American—specialists in their several departments. 600 octavo pages. Illustrated. $2.75. E. B. Treat, Publisher, 5 Cooper Union, New York.

The eighth yearly issue of this handy reference one-volume manual is at hand. In its Alphabetical Index of New Remedies and its Dictionary of New Treatment it richly deserves and perpetuates the well-earned reputation of its predecessors. In this volume its corps of department editors has been largely increased, and important papers upon Thermo-Therapeutics, Electro-Therapeutics, Sanitary Science in city and country, and the Medical Examiner in Life Insurance are features of special interest. It is truly a helpful volume, a *résumé* of the year's progress in medicine, keeping the busy practitioner abreast of the times with reference to the medical literature of the world. While there is a generous increase in size and material, the price remains the same, $2.75.

